# Supplementary material for: Multiple-Reaction Monitoring Tandem Mass Method for Determination of Phenolics and Water-Soluble Vitamins in Eccoilopus formosanus
Source: Molecules. 2020 Aug 10;25(16):3632. doi: 10.3390/molecules25163632 (PMC7464304; doi:10.3390/molecules25163632)
Supplement: Supplementary file 1 [file molecules-25-03632-s001.pdf]

Supplementary materials

Article

Multiple-Reaction Monitoring Tandem Mass Method for Determination of Phenolics and Water-Soluble Vitamins in *Eccoilopus formosanus*

Ho-Shin Huang [1], Hsu-Sheng Yu [2], Chia-Hung Yen [3] and Ean-Tun Liaw [2,*]

1 Food safety center laboratory, Golden Crops Corporation, Yun Lin 640, Taiwan; adinol.huang@gmail.com
2 Department of Food Science, National Pingtung University of Science & Technology, Pingtung 91201, Taiwan; hsyu@mail.npust.edu.tw
3 Department of Biological Science and Technology, National Pingtung University of Science and Technology, Pingtung 91201, Taiwan; chyen0326@mail.npust.edu.tw
* Correspondence: alexliaw@mail.npust.edu.tw; Tel.: +886-8-7740368

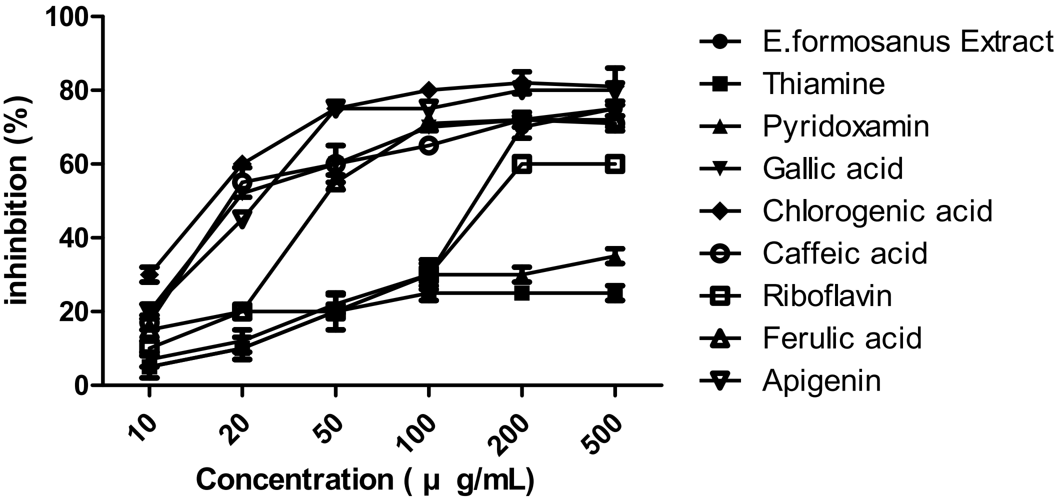

Figure S1. DPPH free radical scavenging ability of *E. formosanus* extract and eight compounds.

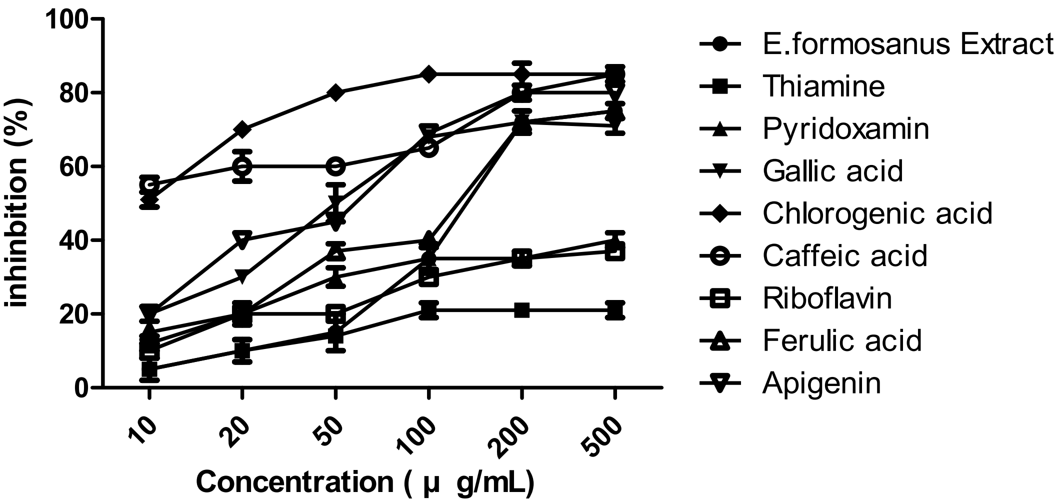

Figure S2. α-Glucosidase inhibition activity of *E. formosanus* extract and eight compounds.
